# Contemporary Themes in Dietary Intake in Rugby Union Players: A Narrative Review

**DOI:** 10.3390/nu16173011

**Published:** 2024-09-06

**Authors:** Charlie J. Roberts, Lewis A. Gough

**Affiliations:** Research Centre for Life and Sport Science (CLaSS), School of Health Sciences, Birmingham City University, Birmingham B15 3TN, UK; charlie.roberts@bcu.ac.uk

**Keywords:** rugby, nutrition, nutrient intake, team sport

## Abstract

Rugby union is an intermittent team sport with variability in body composition and match-play demands between positions which requires careful consideration for individual dietary requirements. While previous reviews have detailed the macronutrient intake in rugby players, none have discussed the further determinants of dietary intake in this population. Therefore, the purpose of the current review was to summarise the current evidence detailing dietary intake in rugby union players, report on contemporary nutritional research themes, and provide recommendations for athletes, nutritionists, and other stakeholders. In total, eighteen articles report on dietary intake in rugby players, with only one of these detailing dietary intake in female athletes. Recent studies have reported on both protein and carbohydrate periodisation practices in rugby union players; however, there is currently limited evidence as to the influence of these on performance, recovery, and well-being. Factors influencing eating patterns, the impact of sports nutritionists on dietary intake, and food consumption in catered and non-catered environments has been explored in isolated studies. Nutrition knowledge levels in rugby players have been reported in several studies; however, the influence this has on dietary intake in rugby players is unknown. Collectively, despite new contemporary themes emerging in the literature concerning dietary intake in rugby players, the studies are isolated; as such, there is limited scope to the translatability of information due to heterogeneity in sex, level of play, and location of participants. Given this, future research should aim to build upon the themes identified in this review in combination to support practitioners working within their specific environments. This will subsequently build towards the generation of rugby-specific recommendations.

## 1. Introduction

Rugby union (“rugby”) is an intermittent team sport whereby players encounter a variety of movement patterns and demands. High-intensity movements including sprints and jumps are interspersed with low-intensity periods of walking or the ball being out of play. Additionally, rugby is a collision sport with contact being “necessary and integral to play” [1]. Collisions in the form of tackles, rucks, mauls, line-outs, and other miscellaneous collisions [2] are common; an average of 156.1 tackles, 116.2 rucks, and 22.0 scrums occur in matches [3], with tackles completed being positively associated with a higher chance of winning in elite rugby [4].

Alongside the variability of movement patterns and demands during a match, positional heterogeneity is observed in rugby. Whilst all players engage in common movements such as ball handling, tackles, walking, and running, the tactical differences between positions may require varying degrees of endurance, speed, and power capacity [5] to be developed for the successful rugby player. Collectively, players can be separated into forwards and backs. Male and female forwards possess greater total body mass (BM), fat free mass (FFM), and fat mass [6,7,8,9], and engage in greater high-intensity activity due to their involvement in events of static exertion [10]. Backs tend to be involved more in periods of intense running [11,12], possess greater high-intensity running ability and sprint velocities [13,14], and cover greater distances during match-play than forwards [10,15]. Despite different positional demands during match-play [5], rugby players in all positions experience large physiological and metabolic stress in the form of increased muscle damage and soreness, and elevations in metabolites indicative of inflammation and structural damage [16,17]. This creates issues for practitioners, as a ‘one-size fits all’ approach cannot be applied to this population.

Whilst inter- and intra-individual differences will be observed based on age, level of play, competitive season, and geography, rugby players observe high training and competition loads. These will influence their nutritional requirements as prolonged periods of inadequate dietary intake increase the risk of injury or illness and are detrimental to performance via losses in training [18]. Globally, it is estimated that elite rugby players participate in ~17 matches per year, with 20% being involved in ≥25 matches. Furthermore, it is estimated that matches contribute to 5–11% of total rugby exposure [19]. Under-15 regional rugby academy rugby players in England engage in between 0.5 ± 1.0 and 1.8 ± 0.8 weekly rugby matches between May and August and September and December. Furthermore, both Under-15 and Under-16 players engage in a collective weekly 10.7 ± 1.2 activity occasions due to matches, training, gym, other sport engagements, and physical education [20]. These demands will influence the specific nutritional requirements of the athlete, demand careful consideration for meal timing, and determine how nutrition support is provided.

Due to high-intensity intervals of activity being interspersed with low-intensity periods of standing and walking over an 80 min match [21], both aerobic and anaerobic energy systems are utilised to ensure sufficient fuel provision; ensuring adequate energy intake is observed is crucial for maintaining the availability of fuel substrates for these energy systems [18]. Athletic performance will likely be compromised in the absence of adequate dietary intake and can result in a multitude of physical, psychological, and mental impairments [22]. Dietary carbohydrates are essential given the high-intensity movements including sprints, jumps, tackles, and large distances covered during match-play. Muscle glycogen is depleted in response to consecutive repeated sprints [23], and commencing matches with low-carbohydrate availability is likely a significant factor in sport performance impairment [18]. Additionally, rugby players require adequate dietary protein consumption to facilitate the repair and remodelling of damaged tissue and enhance structural and functional adaptations [24] with amino acid requirements elevated in response to the variable-intensity movement patterns observed in team sports [25]. Providing specific recommendations to rugby athletes based on best-practice guidelines is difficult given the heterogenous match-play and training demands, body composition requirements, and seasonal variation in training goals [26], with broad ranges of 3–7 g·kg^−1^·day^−1^ for carbohydrates [27] and 1.2–2.0 g·kg^−1^·day^−1^ for protein intake [28,29] provided in the literature.

Previous reviews concerning macronutrient intake patterns in rugby union players [24] and collective team-sport athletes [26] have been published; however, a summary of the multifactorial considerations of food choice and dietary intake is warranted. As such, the purpose of this narrative review is to summarise the current literature concerning dietary intake in rugby union players, the current research themes developed since previous reviews, and provide an updated narrative and practical recommendations relevant to both researchers and practitioners.

## 2. Energy

Rugby is an energetic sport with players experiencing significant physical and metabolic stress in response to training and match-play [16]. Variable training and competition loads, individual body composition goals, and involvement in employment alongside athletic obligations, particularly in sub-elite or amateur players, can result in considerable inter-individual variability in both energy intake and expenditure [30]. As such, ensuring the rugby athlete consumes an energy-sufficient diet can be challenging for the nutrition practitioner. Estimations of habitual energy intake in rugby players are displayed in Table 1.

As the energy demands of rugby extend beyond match-play, multiple groups have sought to quantify energy expenditure across two weeks in professional athletes. Ref. [31] observed a mean daily energy expenditure of 5374 ± 644 kcal·day^−1^ in male professional rugby league players. In a mixed rugby union and league cohort, ref. [32] observed energy expenditures of 4010 ± 744, 4414 ± 688, and 4761 ± 1523 kcal·day^−1^ in U16, U20, and U24 male players. Furthermore, the authors report daily energy expenditures of 62 ± 8, 66 ± 10, and 60 ± 18 kcal·kg^−1^ FFM. Elite female rugby players demonstrated average energy expenditures of 13.51 ± 2.28 MJ·day^−1^ across a 14-day international multi-game tournament [33]. Whilst the demands between the rugby codes differ slightly, the use of gold standard doubly labelled water to quantify energy expenditure in these studies collectively highlights the need for careful consideration of the male and female rugby players’ diet, and the variability of energy requirements within cohorts.

The previous literature has reported that energy expenditure is significantly greater in response to collision-based training when compared to matched non-collision activity across a subsequent 5-day training microcycle [34]. Additionally, when compared to the day before a rugby match, resting metabolic rate was significantly greater a day and three days following rugby match-play [35]. Despite the increased energy cost of collision-based activity, there may be no compensatory consumption of energy in the meals following rugby over non-collision-based activity [36], with such observations appearing similar over extended training periods [37]. During a six-day camp, elite adolescent male rugby players engaged in an average of 340 min of rugby activity daily. Energy expenditure and intake were estimated across 4 days; additional measures of pre and post subjective appetite and ad libitum energy intake were undertaken. The authors observed a significant decrease in ad libitum energy intake following the camp, along with a non-significant daily energy deficit of 213 ± 503 kcal·day^−1^ [37]. Alongside mean resting metabolic rate increases of 231 kcal·day-1 the day after rugby match-play [35], such deficits may result in energy insufficiency over a prolonged period, particularly if the athlete is seeking to maintain or gain lean mass.

Low energy availability occurs when dietary energy intake does not meet the demands for normal physiological and metabolic function after accounting for exercise expenditure [38]. Such a situation may be easily encountered due to the high energetic costs previously observed [31,32], particularly if habitual energy intake is compromised [37]. In developmental rugby players, energy intake was 31 ± 11 kcal·kg^−1^ FFM [39] which is below the proposed threshold of 40 kcal·kg^−1^ FFM for ensuring optimal energy availability. Given that high-level rugby players have previously demonstrated energy expenditure at 60 ± 18 kcal·kg^−1^ FFM in Under-24 rugby players [32], meeting energy requirements should be a priority for rugby players to reduce the risk of physiological, psychological, and behavioural impairments [22] Generally, the evidence suggests most, if not all, rugby players struggle to meet the requirements likely required for health and performance. This raises the need for practitioners to monitor intake and use strategies to increase intake.

## 3. Protein

Amino acid consumption is vital for athletes as the low availability of substrates for repair, remodelling, and synthesis of proteins, and the stimulation of these processes, will limit the extent of adaptation from a structured exercise programme [40]. Following a bout of exercise, dietary amino acid availability is necessary to repair structural myofibrillar proteins and synthesise both mitochondrial proteins and enzyme complexes required for energy metabolism, depending on the exercise modality [41,42]. When exercise is performed with low carbohydrate availability, amino acid oxidation is elevated and the net protein balance negative, suggesting muscle proteins are used to support oxidative metabolism [43]. Additionally, strength and power are important characteristics for those involved in collision sports [12,44]; as such, rugby players engage in resistance training programmes alongside sport-specific training to develop skeletal muscle mass, strength, and power [45,46]. Skeletal mass grows when a positive protein balance is achieved (whereby muscle protein synthesis exceeds breakdown) and an appropriate physiological stimulus is applied [47]. Considering the additional demands placed on amino acid metabolism and the requirement of amino acids as substrates for structural remodelling, repair, and adaptation, athlete-specific recommendations for dietary protein intake have been proposed in the literature. It is generally accepted that recommendations in athletes are greater than sedentary individuals with large ranges of dietary protein being advised [1.2–2.0 g·kg^−1^·day^−1^] [28,29]. As displayed in Table 1, rugby players often report habitual protein intakes at the upper limit of or exceeding the recommendations.

It has been suggested that athletes should aim to consume 0.4 g·kg^−1^ BM of protein on 4–6 eating occasions throughout the day to maximise skeletal muscle accretion, with the hypothesis being that this will maximise both acute responses and chronic adaptations [48]. This is a useable strategy for practitioners to use with athletes as they can use a simple meal-by-meal strategy instead of daily targets that can seem abstract. The previous literature has determined that developing elite rugby union players consume >20 g of protein on multiple occasions daily [3.8 ± 0.9 times per day] [49], corresponding to 0.16 g·kg^−1^ BM for a 120 kg forward or 0.25 g·kg^−1^ BM for an 80 kg back. As such, a relative to BM value will better enable the sports nutritionist to provide a bespoke recommendation for the individual player. Collectively, professional and semi-professional rugby players met the 0.4 g·kg^−1^ BM threshold at the ‘lunch’ and ‘dinner’ eating occasion only [50]. Provincial academy players reported habitually consuming ≥0.4 g·kg^−1^ BM dietary protein per meal at the ‘dinner’ eating occasion only. Following the delivery of a nutrition support intervention guided by a full-time sports nutritionist, only the ‘breakfast’ and ‘post-gym’, which corresponded with breakfast on high-volume days, intakes were greater than the proposed meal threshold [51]. Despite these observations, the average daily protein intakes were within recommendations for all groups. It is important to note that the meal reported values for semi-professional forwards [50] and provincial academy players [51] were at the lower end of recommendations, indicating that some athletes may not be consistently meeting the recommendations.

These data suggest that whilst specific meal intakes may be sub-optimal for muscle accretion [48], average protein intakes were always within or exceeded the total daily recommendations [28,29]. MacKenzie-Shalders and colleagues [52] have demonstrated that increasing protein distribution in total daily protein-matched conditions resulted in no difference in lean mass accretion in developing elite rugby players over six weeks. Given this, it cannot be concluded from the current data that increasing protein distribution in rugby players will confer any physiological benefit provided overall daily recommendations are met. However, encouraging a more micro view of meal-by-meal may still be useful to assist with meeting daily recommendations.

## 4. Carbohydrate

The intermittent sprints, distances covered during matches, and high-intensity movement patterns such as jumps and tackles ensure carbohydrates represent a vital component of the rugby athletes’ diet. Both glucose and dietary fat can be aerobically metabolised; however, the process is slower than anaerobic metabolism [23]. Glucose, either from the blood or glycogen stores, can undergo rapid metabolism via glycolysis allowing for the fast re-synthesis of ATP. During a 30 s sprint, glycolysis may represent ~60% of the ATP produced, with the majority of the remainder from the phosphocreatine energy system [53]. Metabolic analysis immediately following rugby union match-play indicates that glycolysis and gluconeogenesis via protein degradation play major roles in the production of energy due to the increased presence of lactate and alanine [16]. Carbohydrate recommendations for the rugby player will vary based on the daily training volume and level of play. Broadly, intakes between 5–10 g·kg^−1^·day^−1^ are recommended [27,54] with elite athletes engaging in multiple daily training sessions being likely to benefit from consumption levels at the higher end of said recommendations. Numerous authors have detailed the habitual carbohydrate intakes of rugby players, which are presented in Table 1.

Muscle glycogen is a major fuel source for ATP resynthesis during a rugby match, with professional players demonstrating a significant reduction in muscle glycogen content following simulated rugby matches. Sixteen professional players completed a simulated rugby league match and experienced significant vastus lateralis muscle glycogen depletion following either 36 h high-carbohydrate (6 g·kg^−1^·day^−1^; 45 ± 9.5%) or low-carbohydrate (3 g·kg^−1^·day^−1^; 38.2 ± 17.5%) intake [55]. Additionally, ref. [56] reported lower reductions in muscle glycogen concentration in athletes habituated to a 7-day 6 g·kg^−1^·day^−1^ carbohydrate diet following rugby simulation. Athletes consuming carbohydrates immediately following exercise demonstrated a significant glycogen repletion rate (51 ± 47%) compared to when feeding was delayed by two hours (24 ± 49%) following rugby league simulation. Despite being rugby league simulations, the fundamental movements and match-play time [57] are similar enough to extrapolate and suggest rugby union may induce similar levels of depletion. Nonetheless, individual on-pitch demands, other nutrition practices, and variability in movement patterns between games may influence the rates of both pre- and post-glycogen. As glucose is a preferred substrate for neuronal ATP provision [58], carbohydrate ingestion prior to engaging in rugby activity may improve tackling technique [59]. Association football players may benefit from improved skill performance when carbohydrate ingestion occurs both prior to and during activity [60]; whilst the demands are different to rugby, both athletes engage in intermittent sprints and apply decision making during match-play.

Across a seven-day competition week, professional male rugby players appear to periodise carbohydrate intake, with consumption on game day greater than that on all other monitoring days other than game day −2 [61]. Pre-event carbohydrate intake was within the recommended 1–4 g·kg^−1^ BM range for athletes [27] for both forwards (3.0 ± 0.9 g·kg^−1^ BM) and backs (3.0 ± 0.5 g·kg^−1^ BM). Furthermore, post-game carbohydrate intake exceeded the 1.0–1.2 g·kg^−1^ BM recommendation for appropriate refuelling following an event in both forwards (1.5 ± 0.7 g·kg^−1^ BM) and backs (2.1 ± 0.8 g·kg^−1^ BM); however, the importance of this was likely minor given the participants engaged in a “rest and recovery” day on game day +1 [61].

Collectively, rugby players often fail to meet daily best-practice sports nutrition guidelines for carbohydrate intake. Whilst carbohydrate consumption around matches may be sufficient for muscle glycogen depletion based on the limited literature [55], this represents a small sample of professional male rugby league players, and the impact this has on training, recovery, performance, and well-being is unknown.

## 5. Fat

Dietary fat is an essential consideration of the diet for rugby players due to providing abundant substrates for energy provision, essential fatty acids, and facilitating fat-soluble vitamin (A, D, E, K) absorption [62]. Furthermore, dietary fat is important for steroid hormone production with low-fat diets potentially negatively influencing testosterone concentrations [63]. Within energy requirements, athletes are advised to prioritize protein and carbohydrate and consume a minimum of 20–25% total energy intake in the form of dietary fat [62]. As displayed in Table 1, habitual fat intake is presented in numerous studies that report overall energy and macronutrient intakes in rugby players; however, these are likely considered of lower importance than protein and carbohydrate to report due to the inherent importance of protein (lean mass, muscle remodelling, and adaptation) and carbohydrate (substrate for both prolonged and high-intensity performance) for these athletes. Nonetheless, aerobic metabolism contributes significantly to the demands of the rugby player; greater aerobic contributions to energy metabolism during repeated sprints occur as time progresses [64]. As such, the provision of both endogenous and exogenous triglycerides represents a major source of energy provision for the rugby athlete. Supplementation with omega-3 polyunsaturated fatty acids may facilitate a reduction in countermovement jump performance and muscle soreness across a five-week pre-season in elite rugby union players [65]. Nonetheless, the literature concerning dietary fat consumption in rugby players remains limited.

## 6. Diet Quality

Athletes represent a unique population in that specific meal patterns are recommended in relation to timing and nutrient composition to best optimise performance and recovery. Additionally, due to the high energy costs associated with physical activity [66] in certain athletic disciplines, a greater consumption of energy is often warranted. This creates a conundrum considering that many sports, including rugby, also face pressure for optimal body composition and fat level targets. Despite much of the sports nutrition research focusing on daily energy and macronutrient intake and determining optimal timings and quantities of nutrients around training and competition, ensuring a high-quality and nutrient-sufficient diet is of the utmost importance for both athleticism and health [66,67].

Due to the provision of vital micronutrients, fluids and fibre, fruit and vegetable intake may be an indicator of diet quality. Recommendations for fruit and vegetable intake are typically around five servings per day, which varies by country or organisation [68,69]. In a cohort of athletes from various disciplines, fruit (2.4 ± 1.0) and vegetable (3.6 ± 1.1) intake may meet recommendations [66]. The diet quality of adolescent male rugby union players in Australia was assessed via a validated diet quality index [67]. Intakes of iron and calcium, nutrients of importance for developing individuals, were above recommendations. Despite this, whilst median daily servings of fruit were above recommendations in Australia (4.7 ± 5.0 servings; recommendation: 2.0 servings), vegetable intake was lower (1.1 ± 0.5 servings; recommendation: 5.5 servings). Smith and colleagues [70] reported greater daily fruit and vegetable intakes in Under-19 (4.4 ± 1.9 servings) compared to Under-16 rugby players (2.8 ± 1.5 servings); however, both averages do not meet the UK government recommendations of exceeding five daily servings. Additionally, grain intake did not meet recommendations (3.7 ± 2.8 servings; recommendation: 7.0 servings). Inadequate fruit (0.82 ± 0.64) and vegetable (1.3 ± 0.81) daily servings, alongside fibre intake (18.8 ± 6.3 g), have been observed in club rugby players [71]. Fruit and vegetable intake represented 6% of total energy intake in amateur rugby players, with intakes of calcium, vitamin A, and vitamin C below the recommended daily amounts, and low fibre intake was observed [72]. Collectively, the available evidence suggests that sports nutritionists working with rugby players, particularly with youth athletes, should ensure that appropriate support and strategies are provided to facilitate improvements in diet quality.

## 7. Determinants of Dietary Intake and Food Choice in Rugby Players

Quantification of the nutrient intake of rugby players in different environments is important; however, the identification of the factors which influence food dietary choices are of the utmost importance. Practitioners working with athletes must understand why an athlete decides to consume certain foods and beverages, with a variety of psycho-physiological, sociological, and situational factors influencing dietary intake.

Adolescent rugby players identified numerous factors that both enabled and were barriers to following healthy eating patterns [73]. Peers, family, food availability, the media, sports performance, and motivation to perform were identified as simultaneous enablers and barriers; for example, the participants reported that family enabled healthy eating by role-modelling healthy eating, whilst siblings provided temptation. In a mixed professional, semi-professional, and developmental rugby cohort, childhood upbringing, body composition, and sport nutrition knowledge were identified as both enablers and barriers influencing their ability to consume an appropriate nutrient-dense diet or maintain optimal body composition [73]. Additionally, convenience and food security may be significant barriers in high-level athletes [73], particularly those without full professional contracts who are required to navigate multiple obligations without the financial security offered by a full contract [74].

Implementing interventions to facilitate positive dietary behaviour changes in athletes is an important component of the role of a sports nutritionist [75]. Athletes at higher levels of competitive play are more likely to have greater access and exposure to nutrition professionals. Super Rugby athletes relied on the team nutritionist to influence dietary intake whereas provincial and developmental players were influenced by the media and team-mates [73]. Given this, a full-time nutrition support protocol in developmental rugby players who ordinarily have limited access to a sports nutritionist was implemented by [74] to investigate the impact on overall and within-day [51] energy and macronutrient intake over a 4-week intervention period. The delivery of nutrition support was informed by behaviour-change techniques [76] and support was delivered as a group, modified individually for each participant. The results indicated that energy (2492 ± 762 vs. 2614 ± 625 kcal·day^−1^) and protein (1.3 ± 0.4 vs. 1.5 ± 0.3 g·kg^−1^·day^−1^) were statistically greater during the intervention period. Furthermore, there was a significant increase in protein intake at breakfast on low-volume training days, and at AM snack, lunch, and evening snack, whilst PM snack and dinner protein consumption decreased. Despite these findings, it is important to consider whether the changes were practically meaningful given the small overall changes observed in nutrient intake.

During competition, elite athletes are often in catered environments, whereby appropriate food and beverages will be available to the athlete. These environments reduce the financial burden on the athlete, reduce the likelihood of an athlete not being able to source food in a new environment, and will often ensure appropriate dietary choices are present for each individual and their health, cultural, ethical, or taste preferences. Catered environments provide their own challenges that must be considered, such as ensuring over-consumption does not occur due to the buffet style of food delivery, distractions due to eating in larger groups, and unusual or unknown food availability [77]. Elite male rugby forwards consume greater energy (5210 ± 674 vs. 4341 ± 654 kcal·day^−1^), protein (2.8 ± 0.3 vs. 2.3 ± 0.3 g·kg^−1^·day^−1^), and fat (2.1 ± 0.3 vs. 1.5 ± 0.3 g·kg^−1^·day^−1^) in catered vs. non-catered environments. No significant difference was observed in carbohydrate intake; however, the values reported were similar to those observed in elite rugby players in the literature (Table 1). No significant difference between catered or non-catered energy or macronutrient levels in backs was apparent [78].

Nutrition knowledge is described as a modifiable factor that can influence the ability to choose, procure, prepare, and consume appropriate food and meals [79]. Poor nutrition knowledge levels have been observed in male adolescent amateur [80], provincial academy [81], and semi-professional rugby players [82]. Greater nutrition knowledge has previously demonstrated a weak positive relationship with overall diet quality and vegetable consumption in elite athletes [83]; however, the collective information discussed suggests practitioners working with rugby athletes must be flexible and understanding of the individual determinants of food choice, and the environmental factors that can influence these, in their approach to providing nutrition support services. Consideration for the level of play and whether this entails periods of catering, the wider support network available to the athlete, and their personal circumstances all require an individual and holistic approach.

## 8. Directions for Future Research

Future research should aim to build upon the themes identified in this review to allow for the development of recommendations that can be generalised to rugby players globally. Of the studies included in the review, only one published in 2022 examined nutrient intake in female rugby players [84]. Female rugby players demonstrate similar anthropometric [8,85,86] and positional differences for performance [86] to elite male players. Additionally, broadly similar game demands have been demonstrated between female [87,88] and male [89,90] rugby players, indicating a similar need for individual nutritional support and prescription. As such, a greater emphasis on quantifying the dietary and nutrient intakes of female rugby players will allow for sex-specific recommendations to be made.

The literature exploring the determinants of food choice and dietary intake in rugby players is warranted, with the current studies limited to athletes in New Zealand which may not translate to populations in other countries. Given that relationships with food and behaviours related to dietary intake are developed during childhood and adolescence [91], further research into the factors that influence nutrition habits during these formative years is essential. Research concerning the influence of stakeholders such as coaches [92] and parents and/or caregivers [93,94] on dietary choices, particularly in younger athletes, may allow practitioners to better support athletes in their care.

Given the large representation of professional or elite players in the literature, future studies should aim to explore dietary intake and the associated themes in amateur, academy, or semi-professional players due to their unique lifestyle demands such as working and studying concurrently to engaging in training and matches. Future research exploring the influence of nutrient intake in relation to rugby-specific movement patterns would be beneficial, specifically surrounding protein and carbohydrate periodisation.

## 9. Conclusions

This review provides an updated perspective of the literature concerning dietary intake in rugby players. Despite the widespread popularity of the sport and demand for individual nutrition support in rugby players, there is limited literature concerning overall nutrient intake in this population. Collectively, rugby players do not distribute their daily dietary protein intake evenly across meals; however, they tend to consume protein at levels within or exceeding best-practice guidelines. Carbohydrate intake is periodised throughout the day and the week in accordance with the structure of training and the timing of matches. Players at all levels appear not to meet best-practice recommendations for carbohydrates; however, whether this has a negative impact on performance and recovery is unknown. Additionally, various determinants of food intake in rugby players have been identified and poor levels of nutrition knowledge have been observed which may influence dietary choices. Based on the available evidence, an introduction to practical support for nutrition practitioners working with rugby players is presented in Table 2.

**Table 1:** Reported habitual energy and macronutrient intake of rugby union players.

								Absolute				Relative					
Author	Year	Country	Age	Body Mass (kg)	Participants (n)	Level	Sex	Energy (kcal)	Protein (g)	CHO (g)	Fat (g)	Energy (kcal·kg)	Protein (g·kg)	CHO (g·kg)	Fat (g·kg)	Assessment Method	Duration of Assessment
Black et al. [95]	2019	New Zealand	Weight gain: 23.4 ± 3.1 Weight maintenance: 24.6 ± 1.8 Weight loss: 23.6 ± 2.3	106.3 ± 13.3	23	Professional	Male	3875 ± 907					2.1 ± 0.4	3.7 ± 1.2	1.6 ± 0.5	Mixed methods—RFPM and researcher observations	3 days
Bradley et al. [96]	2015	United Kingdom	Forward: 24.4 ± 4.0 Back: 24.2 ± 2.0	Forward: 108.1 ± 7.5 Back: 89.5 ± 5.0	20	Elite	Male	Forward: 14.8 ± 1.9 Back: 13.3 ± 1.9 (MJ)					Forward: 2.5 ± 0.3 Back: 2.6 ± 0.2	Forward: 3.3 ± 0.7 Back: 4.1 ± 0.4	Forward: 1.0 ± 0.3 Back: 1.0 ± 0.3	Dietary recall	2 × 2 training days
Bradley et al. [97]	2015	United Kingdom	Forward: 28.0 ± 2.8 Back: 25.1 ± 3.8	Forward: 110.1 ± 6.2 Back: 93.6 ± 5.9	14	Elite	Male	Forward: 16.6 ± 1.3 Back: 14.2 ± 1.2 (MJ)					Forward: 2.7 ± 0.5 Back: 2.7 ± 0.3	Forward: 3.5 ± 0.8 Back: 3.4 ± 0.7	Forward: 1.4 ± 0.2 Back: 1.4 ± 0.3	Estimated food diary	6 days
Burrows et al. [67]	2016	Australia	16.0 ± 2.0	76.5 ± 10.0	25	Sub-elite	Male	10,372 ± 4974 (KJ)	108.1 ± 75.9	317.0 ± 153	88.5 ± 54.9		1.5 ± 0.9	3.6 ± 2.4		FFQ	
Holway et al. [72]	2024	Argentina	Forward: 28.2 ± 6.8 Back: 26.4 ± 8.1	Forward: 100.3 ± 16.6 Back: 79.8 ± 8.2	57	Amateur	Male	Forward: 2827 (1220–6228) Back: 3682 (2104–7982) (Median/Range)	Forward: 117 (56–329) Back: 136 (47–292) (Median/Range)	Forward: 342 (107–821) Back: 401 (177–828) (Median/Range)	Forward: 104 (33–334) Back: 149 (70–428) (Median/Range)		Forward: 1.1 (0.5–3.6) Back: 0.7–3.5) (Median/Range)	Forward: 3.3 (1.1–8.0) Back: 5.3 (2.3–10.0) (Median/Range)	Forward: 1.0 (0.3–3.7) Back: 1.9 (1.0–4.8) (Median/Range)	Dietary recall	1 day
Hitendre et al. [82]	2022	United Kingdom	23.0 (21.5, 27.5) (Median/IQR)	96.6 ±10.8	24	Semi-professional	Male	2472 (1767, 3374) (Median/IQR)	139.8 ± 51.1	266 (199, 341) (Median/IQR)	97.0 (68.3, 150.3) (Median/IQR)	26.3 ± 9.2	1.4 ± 0.4	3.0 (2.0, 3.0)		FFQ	
Hudson et al. [16]	2021	United Kingdom	20.0 ± 2.7	102.5 ± 13.7	7	Professional	Male	3323 ± 630					2.4 ±0.3	3.2 ± 0.4		RFPM	7 days
Imamura et al. [98]	2013	Japan	Forward: 19.5 ± 0.9 Back: 19.5 ± 1.0	Forward: 87.3 ± 8.9 Back: 72.6 ± 7.4	34	Collegiate	Male	Forward: 3579 ± 848 Back: 2963 ± 111	Forward: 92.7 ± 22.3 Back: 79.9 ± 31.5	Forward: 567.0 ± 160.1 Back: 457.4 ±192.2	Forward: 91.5 ± 25.0 Back: 77.2 ± 30.8					FFQ	1–2 months
MacDougall et al. [71]	2015	USA	30.2 ± 1.1		15	Collegiate	Male	2378 ± 126					1.7 ± 0.8	3.4 ± 1.1		Dietary recall	3 days
MacKenzie et al. [49]	2015	Australia	20.5 ± 2.3	100.2 ± 13.3	25	Developing elite	Male	3250 ± 869	211.0 ± 62.0	352.0 ± 115.0	101.0 ± 34.0		2.2 ± 0.7	3.6 ± 1.3	1.1 ± 0.5	Food record	7 days
Posthumus et al. [61]	2021	New Zealand	27.6 ± 2.8	103.0 ± 13.6	34	Professional	Male	Forward: 4606 ± 719 Back: 3761 ± 618	Forward: 280 ± 39 Back: 220 ± 37	Forward: 399 ± 77 Back: 340 ± 59	Forward: 210 ± 43 Back: 169 ± 41	Forward: 40.5 ± 7.2 Back: 41.9 ± 7.2	Forward: 2.5 ± 0.4 Back: 2.4 ± 0.5	Forward: 3.5 ± 0.8 Back: 3.7 ± 0.7	Forward: 1.8 ± 0.4 Back 1.8 ± 0.5	RFPM	7 days
Posthumus et al. [78]	2022	New Zealand	Forward: 28.2 ± 2.9 Back: 28.5 ± 3.2	Forward: 115.0 ± 6.8 Back: 93.2 ± 7.6	12	Professional	Male	Forward (catered): 5210 ± 674 Forward (non-catered): 4341 ± 654 Back (catered): 3952 ± 765 Back (non-catered): 3445 ± 610	Forward (catered): 318 ± 33 Forward (non-catered): 260 ± 29 Back (catered): 223 ± 46 Back (non-catered): 188 ± 11	Forward (catered): 408 ± 85 Forward (non-catered): 411 ± 89 Back (catered): 328 ± 65 Back (non-catered): 317 ± 75	Forward (catered): 244 ± 34 Forward (non-catered): 183 ± 47 Back (catered): 183 ± 47 Back (non-catered): 149 ± 50	Forward (catered) 45.8 ± 7.2 Forward (non-catered): 38.2 ± 6.6 Back (catered): 42.8 ± 8.4 Back (non-catered): 37.6 ± 5.9	Forward (catered): 2.8 ± 0.3 Forward (non-catered): 2.3 ± 0.3 Back (catered): 2.4 ± 0.5 Back (non-catered): 2.0 ± 0.1	Forward (catered): 3.6 ± 0.9 Forward (non-catered): 3.6 ± 0.8 Back (catered): 3.5 ± 0.6 Back (non-catered): 3.4 ± 0.7	Forward (catered): 2.1 ± 0.3 Forward (non-catered): 1.5 ± 0.3 Back (catered): 2.0 ± 0.5 Back (non-catered): 1.6 ± 0.6	RFPM	Catered: 7 days Non-catered: 7 days
Potgieter et al. [99]	2014	South Africa	N/A *	N/A *	11	Collegiate	Male					45.4 ± 9.0	2.4 ± 0.7	4.3 ± 0.4	1.9 ± 0.5	Mixed-methods—food record and 24 h recall	7 days
Roberts et al. [50]	2022	New Zealand	Professional: 31.4 ± 3.0 Semi-professional forward: 24.2 ± 3.5 Semi-professional back: 24.4 ± 4.7	Professional: 104.9 ± 12.0 Semi-professional forward: 114.3 ± 8.2 Semi-professional back: 93.8 ± 6.2	Professional: 10 Semi-professional forward: 19 Semi-professional back: 6	Professional and semi-professional	Male		Professional: 203.7 ± 68.1 Semi-professional forward: 151.5 ± 64.6 Semi-professional back: 178.7 ± 47.3				Professional: 2.0 ± 0.6 Semi-professional forward: 1.4 ± 0.5 Semi-professional back: 1.9 ± 0.5			RFPM	7 days *
Roberts et al. [74]	2022	New Zealand	20.5 ± 1.6	102.4 ± 18.2	10	Provincial academy	Male	2492 ± 762	131.1 ± 41.8	261.7 ± 101.3	95.8 ± 34.7	24.4 ± 7.5	1.3 ± 0.4	2.6 ± 1.0	0.9 ± 0.3	RFPM	Observation: 12 days
Smith et al. [70]	2016	United Kingdom	Under 16: 15.8 ± 0.5 Under 19: 18.1 ± 0.8	Under 16: 83.7 ± 12.3 Under 19: 91.4 ± 13.4	52	Elite academy	Male	Under 16: 3269 ± 766 Under 19: 3412 ± 670	Under 16: 155.4 ± 56.4 Under 19: 210.7 ± 46.7	Under 16: 392.2 ± 108.2 Under 19: 416.2 ± 107.2	Under 16: 112.3 ± 34.2 Under 19: 111.8 ± 34.8	Under 16: 37.9 ± 10.4 Under 19: 38.2 ± 9.8	Under 16: 1.9 ± 0.6 Under 19: 2.3 ± 0.5	Under 16: 4.8 ± 1.1 Under 19: 4.7 ± 1.4	Under 16: 1.4 ±0.5 Under 19: 1.3 ± 0.5	Food record	4 days
Traversa et al. [84]	2022	Canada	20.5 ± 0.4	74.9 ± 2.9	15	Varsity	Female	2158 ± 87	99.6 ± 4.6	246.9 ± 12.2	91.0 ± 4.6	38.6 ± 2	1.4 ± 0.1	3.4 ± 0.2	1.2 ± 0.1	Weighed food diary (online application)	7 days
Zyla et al. [100]	2014	Poland	Forward: 24.0 ± 4.3 Back: 22.0 ± 1.7	Forward: 92.6 ± 14.7 Back: 80.8 ± 8.6	44	Amateur	Male	3613 ± 943	157.7 ± 51.6	404.5 ± 128.4	158.3 ± 52.1		1.9 ± 0.7	4.8 ± 1.7	1.9 ± 0.7	Dietary recall	3 days

* Age and body composition values in the study are provided for n = 18 participants whereas nutrient intake data is provided for n = 11 participants.

**Table 2:** Introduction to practical support for practitioners working with rugby players.

Factor	Advisory	Methods to Meet Advisory	Level of Evidence	Directions for Future Research
Energy	Ensure energy intake meets the demands of the sport. The current literature suggests that rugby players have high energy demands but the levels of intake required to maintain energy balance may not be sufficient.	Education of athletes Delivery of education workshops around unique energetic demands of rugby athletes, with consideration for positional variability Provision of infographics for personal use and in food areas within the rugby environment App-based education and communication (e.g., WhatsApp) Engagement with athletes around meals in catered environments Education of stakeholders Delivery of education workshops and infographics around the unique energetic demands of rugby athletes, with considerations for positional variability Provision of tailored shopping lists Provision of food and batch-tested supplements Explore canteen implementation and transformations Food parcels, particularly for academy players with various rugby-specific and external obligations, to ensure meeting energy requirements Outsource food delivery and preparation Appropriate estimation of resting metabolic rate and energy expenditure when direct determination of energy expenditure is not available Utilise a rugby-specific resting metabolic rate estimation equation [101]	Moderate	Quantify energy intake in female rugby players Investigate barriers and facilitators to enable the meeting of energy demands
Protein	Ensure per-meal protein intake is in-line with best-practice sports nutrition guidelines in the literature (0.4 g·kg^−1^ BM of protein on 4–6 eating occasions throughout the day; Schoenfeld and Aragon, 2018)	Education of athletes Delivery of education workshops providing information and recommendations for protein intake based on specific athletes’ body mass and activity levels, with consideration for within-day protein periodisation Provision of infographics for personal use and in food areas within the rugby environment App-based education and communication (e.g., WhatsApp) Engagement with athletes around matches, training, and in catered environments Education of stakeholders Delivery of education workshops and infographics providing information and recommendations for within-day and overall protein intake based on specific athletes’ body mass and activity levels Provision of tailored shopping lists, with specific considerations for individual athlete body mass and activity levels to ensure optimal carbohydrate availability Provision of food and batch-tested supplements Protein stations in changing rooms to ensure availability around matches and training sessions Provision of batch-tested supplements for athletes to introduce into the home environment	Low	Quantify protein intake in female rugby players Determine the impact of periodising within-day protein intake on rugby-specific movement patterns, performance, body composition, and recovery
Carbohydrate	Ensure daily carbohydrate is in-line with best-practice sports nutrition guidelines in the literature (5–10 g·kg^−1^·day^−1^; [27], with considerations for food volume due to observed body mass variability.	Education of athletes Delivery of education workshops providing information and recommendations for carbohydrate intake based on specific athletes’ body mass and activity levels, with consideration for within-day and between-day carbohydrate periodisation Provision of infographics for personal use and in food areas within the rugby environment App-based education and communication (e.g., WhatsApp) Engagement with athletes around meals in catered environments Education of stakeholders Delivery of education workshops and infographics providing information and recommendations for carbohydrate intake based on specific athletes’ body mass and activity levels, with considerations for carbohydrate periodisation Provision of tailored shopping lists, with specific considerations for individual athlete body mass and activity levels to ensure optimal carbohydrate availability Provision of food and batch-tested supplements Explore canteen implementation and transformations Food parcels, particularly for academy players with various rugby-specific and external obligations, to ensure meeting carbohydrate requirements Outsource food delivery and preparation	High	Quantify carbohydrate intake in female rugby players Carbohydrate periodisation in young academy athletes Determine the impact of periodising carbohydrate intake (between-day and within-day) on rugby-specific movement patterns, performance, body composition, and recovery
Diet quality	Support increases in fruit and vegetable intake in youth athletes	Education of athletes Delivery of education workshops providing information and recommendations for optimising diet variety and quality Provision of infographics for personal use and in food areas within the rugby environment App-based education and communication (e.g., WhatsApp) Engagement with athletes around meals in catered environments Education of stakeholders Delivery of education workshops and infographics providing information and recommendations for optimising diet variety and quality Provision of tailored shopping lists Provision of food and batch-tested supplements Explore canteen implementation and transformations Food parcels, particularly for academy players with various rugby-specific and external obligations Outsource food delivery and preparation	Low	Quantification of fruit and vegetable intake in elite rugby players Quantification of fibre intake
Determinants of food choice	Ensure stakeholders such as coaches, support staff, partners, and caregivers are able to appropriately support food choice provision. Increase nutrition knowledge in athletes and stakeholders.	Education of athletes Delivery of education workshops with specific focus on techniques to improve dietary intake and quality Provision of infographics for personal use and in food areas within the rugby environment Engage in one-to-one discussions with athletes to understand the unique determinants of food choice in environments other than the specific rugby environment (e.g., home, work or school, during travel) App-based education and communication (e.g., WhatsApp) Engagement with athletes around meals in catered environments Education of stakeholders Engage in one-to-one discussions with parents and caregivers to understand the unique determinants of food choice in environments other than the specific rugby environment (e.g., home, work or school, during travel) Delivery of education workshops and infographics providing information on techniques to improve dietary intake and quality Provision of tailored shopping lists Where practical, ensure greatest availability of sports nutrition practitioners within the club environment Implementing strategies to improve dietary practices in rugby players via regular communication and monitoring of players, and delivery of educational materials to both players and stakeholders may be unfeasible without appropriate practitioner investment [74] Utilise evidence base to structure systematic approach to nutrition support provision	Low	Greater geographic variability in qualitative understanding of determinants of food choice in rugby players Determination of barriers and facilitators to meeting generic and sport-specific nutrition guidelines for athletes and stakeholders Further quantification of nutrition knowledge in athletes and stakeholders

## Data Availability

Not applicable.
